# A Digital Coach (E-Supporter 1.0) to Support Physical Activity and a Healthy Diet in People With Type 2 Diabetes: Acceptability and Limited Efficacy Testing

**DOI:** 10.2196/45294

**Published:** 2023-07-28

**Authors:** Eclaire A G Hietbrink, Wendy Oude Nijeweme-d’Hollosy, Anouk Middelweerd, Annemieke A J Konijnendijk, Laura K Schrijver, Anouk S ten Voorde, Elise M S Fokkema, Gozewijn D Laverman, Miriam M R Vollenbroek-Hutten

**Affiliations:** 1 Department of Biomedical Signals and Systems University of Twente Enschede Netherlands; 2 Department of Internal Medicine/Nephrology Ziekenhuisgroep Twente (ZGT) Almelo Netherlands; 3 Office of Research and Innovation Santeon Utrecht Netherlands; 4 Board of Directors Medisch Spectrum Twente (MST) Enschede Netherlands

**Keywords:** eHealth, type 2 diabetes, physical activity, nutrition, lifestyle change, acceptability, limited efficacy, formative evaluation, mobile phone

## Abstract

**Background:**

A healthy lifestyle, including regular physical activity and a healthy diet, is increasingly part of type 2 diabetes (T2D) management. As many people with T2D have difficulty living and maintaining a healthy lifestyle, there is a need for effective interventions. eHealth interventions that incorporate behavior change theories and tailoring are considered effective tools for supporting a healthy lifestyle. The E-Supporter 1.0 digital coach contains eHealth content for app-based eHealth interventions and offers tailored coaching regarding physical activity and a healthy diet for people with T2D.

**Objective:**

This study aimed to assess the acceptability of E-Supporter 1.0 and explore its limited efficacy on physical activity, dietary behavior, the phase of behavior change, and self-efficacy levels.

**Methods:**

Over a span of 9 weeks, 20 individuals with T2D received daily motivational messages and weekly feedback derived from behavioral change theories and determinants through E-Supporter 1.0. The acceptability of the intervention was assessed using telephone-conducted, semistructured interviews. The interview transcripts were coded using inductive thematic analysis. The limited efficacy of E-Supporter 1.0 was explored using the Fitbit Charge 2 to monitor step count to assess physical activity and questionnaires to assess dietary behavior (using the Dutch Healthy Diet index), phase of behavior change (using the single-question Self-Assessment Scale Stages of Change), and self-efficacy levels (using the Exercise Self-Efficacy Scale).

**Results:**

In total, 5 main themes emerged from the interviews: perceptions regarding remote coaching, perceptions regarding the content, intervention intensity and duration, perceived effectiveness, and overall appreciation. The participants were predominantly positive about E-Supporter 1.0. Overall, they experienced E-Supporter 1.0 as a useful and easy-to-use intervention to support a better lifestyle. Participants expressed a preference for combining E-Supporter with face-to-face guidance from a health care professional. Many participants found the intensity and duration of the intervention to be acceptable, despite the coaching period appearing relatively short to facilitate long-term behavior maintenance. As expected, the degree of tailoring concerning the individual and external factors that influence a healthy lifestyle was perceived as limited. The limited efficacy testing showed a significant improvement in the daily step count (*z*=−2.040; *P=*.04) and self-efficacy levels (*z*=−1.997; *P=*.046) between baseline and postintervention. Diet was improved through better adherence to Dutch dietary guidelines. No significant improvement was found in the phase of behavior change (*P=*.17), as most participants were already in the maintenance phase at baseline.

**Conclusions:**

On the basis of this explorative feasibility study, we expect E-Supporter 1.0 to be an acceptable and potentially useful intervention to promote physical activity and a healthy diet in people with T2D. Additional work needs to be done to further tailor the E-Supporter content and evaluate its effects more extensively on lifestyle behaviors.

## Introduction

### Background

Type 2 diabetes (T2D) is 1 of the 4 most prevalent noncommunicable diseases worldwide and has a major impact on the health and well-being of individuals [1,2]. In 2021, approximately 536.3 million adults were living with diabetes mellitus, and this number is predicted to rise to 783.2 million in 2045 [3]. A healthy lifestyle is of utmost importance in the management of T2D and its complications [4-7]. Therefore, obesity, unhealthy diet, and sedentary lifestyle are important treatment targets in T2D [8-10]. Physical activity increases insulin sensitivity [11]; stimulates weight loss; and improves blood pressure, lipoprotein profile, vascular health, and general fitness [12,13]. In addition, a healthy diet (ie, rich in whole grains, fruits, vegetables, legumes, and nuts and lower in refined grains, red and processed meats, and sugar-sweetened beverages) improves glycemic control and blood lipid profiles in people with T2D [14]. Therefore, lifestyle modification is a potential strategy for managing or even reversing T2D. However, most people with T2D experience difficulties in meeting the guidelines on physical activity and a healthy diet [15,16].

To support people with T2D in adopting a healthy lifestyle, several lifestyle interventions have been developed, which have clearly shown that such interventions can achieve the reversal or remission of T2D [4-7,17,18]. However, a disadvantage of most lifestyle interventions is that many are offered by professionals, which makes them time and cost intensive. With the growing diabetes population, capacity issues, and limited financial resources available, providing lifestyle guidance to all people with T2D is not feasible via face-to-face programs alone [19,20]. Therefore, remote or blended-care solutions are needed to make lifestyle guidance accessible to all people with T2D. eHealth (ie, the use of technology to support health, well-being, and health care [21]) can play a major role in this [22], as they are accessible at all times and are less intensive regarding effort, time, and cost than face-to-face programs [23,24]. Furthermore, eHealth can be used to self-monitor lifestyle behaviors and enables more continuous support in daily life through tailored feedback [24,25]. Moreover, extensive research showed that eHealth has the potential to improve physical activity levels [26-28] and compliance with dietary guidelines [29,30], resulting in improved health outcomes, such as perceived fitness, body weight, blood pressure, or glycemic control [29-32]. However, the effectiveness of eHealth interventions differs according to the intervention [28,33]. This variability in effectiveness can be explained by the great diversity within these interventions in the use of potentially effective elements, such as the use of behavior change theory (eg, social cognitive theory [34]) or the degree of tailoring to the user [35].

Several theories of health behavior have been developed to explain health behaviors and to guide the development of behavior change interventions. Research showed that interventions based on a behavior change theory are more effective than interventions that are not based on a theory [36-39]. In addition, using behavior change techniques (BCTs; active components of an intervention designed to alter or redirect causal processes that regulate behavior [40]) increases the likelihood of an intervention being effective [41,42]. To illustrate, most interventions that reported significant changes in physical activity, diet, or health outcomes combined BCTs as *self-monitoring*, *goal setting*, *feedback on behavior*, or *review of behavioral goals* [29,43]. Nevertheless, many eHealth interventions lack the optimal use of effective behavioral change theory and techniques [44-47]. Furthermore, several studies have shown that tailored eHealth interventions are more effective in promoting healthy behaviors and user engagement than generic (ie, nontailored) interventions [33,35,39,48-50]. Moreover, dynamically tailored interventions (ie, feedback is based on iterative assessment) have shown larger effect sizes and better long-term effects than static tailored interventions (ie, where all feedback are based on a single baseline assessment) [51,52].

Currently, eHealth interventions aimed at improving lifestyle behavior that integrate behavior change theory and dynamic tailoring are scarce in the Dutch market. In this light, we are developing an eHealth intervention to support a healthy lifestyle in people with chronic diseases, called *E-Supporter*, through a systematic and iterative approach. In the first iteration, we developed *E-Supporter 1.0*, in which both behavior change theory and dynamic tailoring are embedded to support people with T2D in improving physical activity levels and nutritional behavior. On the basis of if-then rules, E-Supporter 1.0 offers three types of intervention options: (1) motivational messages, (2) behavioral feedback, and (3) tailor-made supportive exercises to overcome barriers to achieving goals. E-Supporter 1.0 is described in detail elsewhere [53].

### Objectives

Developing E-Supporter is an iterative and dynamic process in which the intervention is improved step by step. Therefore, an interim evaluation of the intervention program is a necessary step to examine whether the intervention is appropriate for further development or testing and to formulate points for improvement for subsequent development iterations [21,54,55]. Accordingly, it may be worthwhile to explore some aspects of feasibility [55], such as users’ overall experience with the intervention (ie, acceptability) or whether the intervention is potentially successful among the target population (ie, limited efficacy). As we do not have insight into the aforementioned concepts regarding E-Supporter 1.0, the aims of this study were to (1) evaluate the patient’s acceptability of E-Supporter 1.0 and (2) explore its limited efficacy on physical activity, dietary behavior, the phase of behavior change, and self-efficacy levels.

## Methods

### Overview

**Figure 1:**
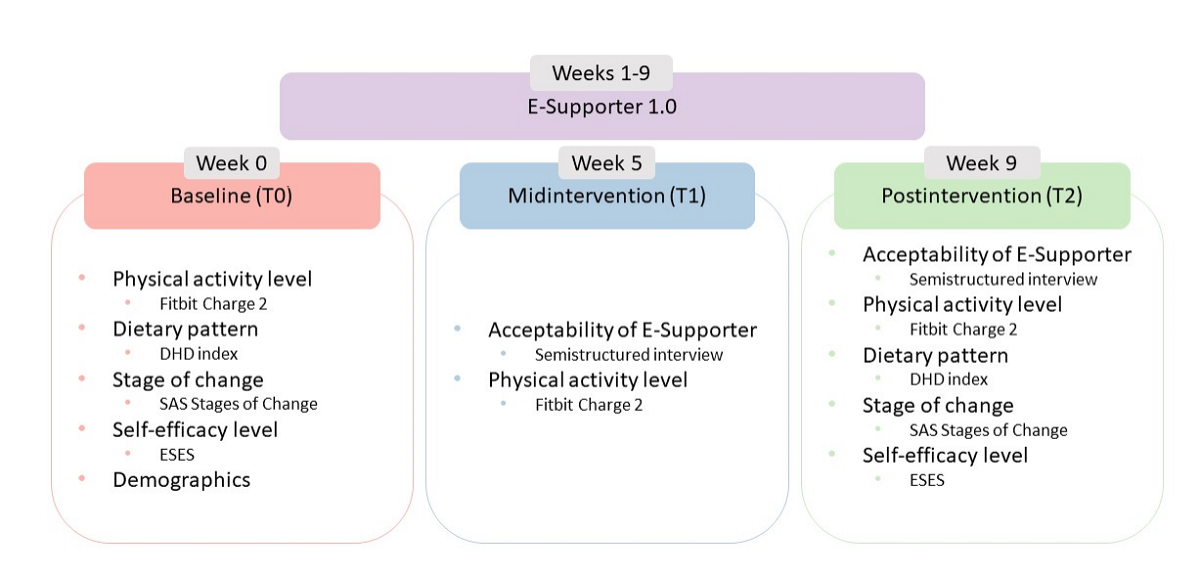
Study design. DHD15 index: Dutch Healthy Diet index [56]; ESES: Exercise Self-Efficacy Scale [57]; SAS Stages of Change: Single-question Self-Assessment Scale Stages of Change [58].

A mixed methods longitudinal study was conducted to evaluate the acceptability and limited efficacy [55] of E-Supporter 1.0 on physical activity, diet, phase of behavior change, and self-efficacy. The study included 3 data collection points: baseline assessment (T0), midintervention assessment (T1), and postintervention assessment (T2; Figure 1).

#### Intervention Description

The E-Supporter 1.0 coaching content aimed to encourage people with T2D to adopt a healthier lifestyle regarding light to moderate to vigorous physical activities or better adherence to the Dutch dietary guidelines. E-Supporter 1.0 consisted of coaching content that can be integrated into various app-based eHealth interventions. More detailed information about the design of E-Supporter 1.0 can be found elsewhere [53].

#### Intervention Targets

On the basis of behavior change theories, several behavioral intervention targets (ie, determinants) are addressed in the content of E-Supporter 1.0. These intervention targets were selected based on the Health Action Process Approach [59] and theories that elucidate behavioral maintenance, including Rothman’s theory of maintenance [60] or Marlatt’s relapse prevention theory [61]. The intervention approach recognized three distinct phases of behavior change: (1) an intentional phase to form intentions to adopt a healthy behavior, (2) an action phase to transform intentions into actual behavior change, and (3) a maintenance phase to support the persistence of behavior change in the long term.

**Figure 2:**
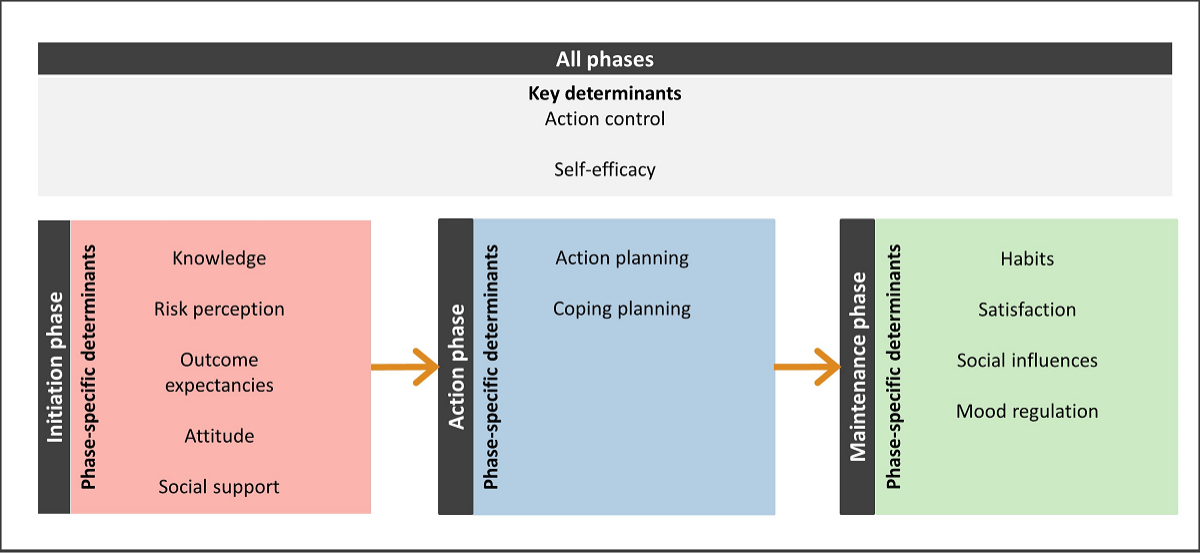
Intervention targets of E-Supporter 1.0.

The intervention targets consisted of 2 key determinants: action control and self-efficacy. E-Supporter 1.0 further covered phase-specific determinants for all the 3 phases, such as outcome expectancies, coping planning, and habit formation (Figure 2). BCTs (eg, *self-monitoring of behavior*, *information about health consequences*, and *prompts and cues*) were included in the intervention content to address the key and phase-specific determinants.

#### Intervention Options

##### Overview

E-Supporter 1.0 consisted of goal setting options (ie, step goals, cycling goals, or nutritional goals) and 3 types of intervention options. The intervention options included the type of behavior change support that could be used at any decision point (ie, points in time at which an intervention decision must be made) [25]. The intervention options in E-Supporter 1.0 comprised (1) motivational messages, (2) feedback on behavior, and (3) tailor-made psychological exercises to overcome barriers to goal achievement. Intervention options were tailored based on if-then rules to the variables of behavior goal, phase of behavior change, type of chronic disease, time of day, and goal achievement. To provide insight into and feedback on current lifestyle behavior, the E-Supporter content could be used in combination with self-monitoring tools.

In this study, the content of E-Supporter was examined via text messaging and email without being integrated into an app. Psychological exercises are normally offered through a conversational agent and can therefore only be offered if it is integrated into an app. Hence, only motivational messages (via text messaging) and feedback on behavior (via email) were evaluated in this study.

##### Motivational Messages

Motivational messages were sent a maximum of twice a day and could be tailored to the phase of behavior change, type of chronic disease, time of day, and type of behavioral goal. The content of the motivational messages was based on the behavioral determinants and the corresponding BCTs in E-Supporter 1.0. Motivational messages included, but were not limited to, knowledge about guidelines for physical activity or nutrition, information about setting achievable goals, or making action and coping plans.

##### Feedback

Feedback was tailored to goal achievement and was provided weekly after the last goal assessment moment of the week. Feedback was given regarding whether they achieved their goal, consisting of both descriptive and evaluative feedback. Everyone received feedback on the number of days the goal had been achieved in the past week. If individuals realized a great deal of their goal or even their full goal, they received a compliment.

### Participants

We aimed to recruit 20 people with T2D who participated in the observational Diabetes and Lifestyle Cohort Twente (DIALECT) study [62] in Ziekenhuisgroep Twente (ZGT) Almelo, the Netherlands. Participants were eligible for the study if they met the following criteria: (1) diagnosed with T2D, (2) in possession of a smartphone, (3) Dutch speaking, and (4) signed an informed consent form. Participants were excluded if they were unable to open or read text messages or email.

### Procedures

In total, 3 rounds of assessments were conducted: at baseline (T0), midintervention (T1), and postintervention (T2). Participants could work on either physical activity or diet. Participants in the physical activity module wore a Fitbit Charge 2 throughout the intervention period. Participants in the nutritional module could maintain an electronic food diary for the entire intervention period.

Participants were informed of the study during a face-to-face appointment for the DIALECT study. Participants of the DIALECT study who were interested in participating in this study received detailed information about the study. The intake took place during the second appointment for the DIALECT study at the ZGT Hospital. During the intake, participants could ask questions about their participation in the study and signed an informed consent form. The researcher assisted the participants by installing either the Fitbit Charge 2 and Fitbit app or the Diameter app [63,64] to maintain the electronic food diary.

To complete the baseline measurements, participants in the physical activity module were asked to wear the Fitbit Charge 2 for 1 week to assess physical activity levels at baseline. Participants in the nutritional module were asked to complete a questionnaire on their dietary habits. In addition, all participants were asked to complete 2 questionnaires to determine their phase of behavior change and self-efficacy level at baseline.

Thereafter, participants set either a step goal or nutritional goal according to the Dutch Health Eating Guidelines (eg, eating 2 pieces of fruit daily) [65]. The goals gradually became more difficult over the weeks (eg, a higher step goal each week). Participants used E-Supporter 1.0 for 9 weeks. Motivational messages were tailored based on the baseline questionnaires regarding the phase of behavior change and self-efficacy level. The feedback was tailored based on the assessed behavior and goal achievement. Data to provide feedback were collected using Fitbit Charge 2 for the physical activity module and daily ecological momentary assessments for the nutritional module. Through the ecological momentary assessments, participants in the nutritional module were asked every day whether they had achieved their goal the previous day. Daily motivational messages were sent as text messages, and feedback was sent via email. After the feedback on goal achievement, the mail contained the behavioral goal for the next week.

During midintervention and postintervention, the interviews to assess acceptability were conducted by telephone and were audio recorded. The interviews lasted between 15 and 60 minutes, with an average of 25 minutes, and were conducted by AStV, EMSF, and LKS. Physical activity levels were assessed at both midintervention and postintervention for the participants in this module. Diet was only assessed postintervention. In week 9, participants were invited to complete the web-based postintervention questionnaires regarding the phase of behavior change and self-efficacy levels.

### Data Collection and Analysis

#### Overview

Statistical analysis was performed using ATLAS.ti (version 8) and SPSS (version 25; IBM Corp). Descriptive analyses were conducted for all quantitative variables (means or medians, SDs or IQRs, and frequencies). A significance level of *P*<.05 was used for all comparisons.

#### Acceptability

We operationalized acceptability as users’ overall experience with E-Supporter 1.0, including perceived effectiveness, ease of use, and satisfaction with the experience [55]. In total, 2 semistructured interviews at midintervention and postintervention were conducted to assess the acceptability of E-Supporter 1.0. The interviewers followed an interview schedule developed by the authors to conduct the interview (Multimedia Appendix 1). The participants were first given a brief introduction about the purpose of the interview. Subsequently, participants were asked about their overall appreciation of the intervention, their opinion about the motivational messages, and their opinion about the weekly feedback and to express their appreciation in a grade for motivational messages, feedback, and the entire intervention on a scale of 1 (very poor) to 10 (excellent). Participants were asked a series of open-ended questions. An example of an open-ended question was, “What do you think of the content of the text messages?” The interview guide included follow-up questions to elicit relevant experiences.

The interviews were transcribed nonverbatim. Inductive thematic analysis [66] was used to analyze the transcripts. Each text fragment that seemed to provide an important perspective on the acceptability of our intervention was assigned a descriptive code. The codes were then iteratively specified to map the underlying themes. Transcripts were independently coded by 2 researchers. The assigned codes were compared, and in cases of disagreement, the researchers discussed until a consensus was reached. Themes were developed, defined, and where necessary, and refined from the specified codes.

#### Physical Activity

Physical activity levels were measured by extracting the mean daily step count per day, as assessed by Fitbit Charge 2 [67]. The Fitbit is a triaxial accelerometer that measures daily step count, minutes spent per activity level, stairs climbed, calories burned, distance traveled, heart rate, and sleep patterns. In this study, only daily step count was used. To self-monitor physical activity for the intervention period, the participants were requested to wear the Fitbit daily. We reported the step count as the median (IQR). A Wilcoxon signed-rank test [68] was performed to examine differences in physical activity levels between baseline versus midintervention and baseline versus postintervention.

#### Dietary Habits

Dietary intake was assessed using a validated, semiquantitative Food Frequency Questionnaire (FFQ) [69]. For each food item, the FFQ contained questions regarding the frequency (in times per day, week, or month), portion size (in natural or household measures), and preparation methods. Daily nutrient intake per food item (grams per day) was determined using the Dutch Food Composition Table [70]. The Dutch Healthy Diet index (DHD15 index) score was used to assess adherence to the Dutch dietary guidelines in 1 score based on 15 components of a healthy diet [56]. FFQ data were categorized into DHD15 index food groups. The DHD15 index assigned a score between 0 and 10 points to every component. The assignment of the scores has been described elsewhere [56]. Adherence to nutritional guidelines was expressed by summing all components into a score on a scale of 0 (not adherent) to 120 (fully adherent) per participant. The maximum score is normally 150. However, because of the limitations of the FFQ used in this study in accurately estimating the intake of coffee, salt, and whole-grain products, these components were excluded from the analysis. The difference in the DHD15 index score was calculated between baseline and postintervention and presented as absolute and percent improvement.

#### Self-Efficacy

To measure self-efficacy, the Exercise Self-Efficacy Scale (ESES) was administered (Multimedia Appendix 2 [57,71]) at baseline and postintervention. The ESES consists of 10 items on self-confidence about physical activity. For participants in the nutritional module, we used a modified version of the ESES by Fokkema [71] to determine self-efficacy regarding a healthy diet. The assessment was based on a 4-point Likert scale, containing the anchors “1=not at all true,” “2=rarely true,” “3=sometimes true,” and “4=always true” [57]. Self-efficacy was categorized as a continuous variable by summation of the scores on the 10 items of the ESES questionnaire. The minimum and maximum scores were 10 and 40, respectively, with a higher score indicating a higher level of self-efficacy. Self-efficacy scores are presented as median (IQR), and a Wilcoxon signed-rank test was performed [68]. Generally accepted cutoff values were used to indicate the level of self-efficacy; a total score of <29 indicated low self-efficacy, and a total score of ≥29 indicated high self-efficacy [72].

#### Phase of Behavior Change

We used the single-question Self-Assessment Scale (SAS) Stages of Change (Multimedia Appendix 3 [58,71]) to measure the phase of behavior change at baseline and postintervention. For participants in the nutritional module, we used a modified version of the SAS Stages of Change by Fokkema [71] to determine the phase of behavior change regarding a healthy diet. The SAS Stages of Change measure the phase of behavior change based on a single question by asking the extent to which someone participated in healthy behavior. The possible answer options related to 5 phases of behavior change as described in the transtheoretical model [73] and were used to categorize the phase of behavior change as an ordinal variable from 1 to 5 (1=maintenance, 2=action, 3=preparation, 4=contemplation, and 5=precontemplation) [74]. The 5 answer options were traced to the 3 phases of behavior change tailored to E-Supporter 1.0. Participants who chose answer option 3, 4, or 5 were included in the initiation phase; participants who answered option 2 were included in the action phase; and participants who chose answer option 1 were included in the maintenance phase. A related sample marginal homogeneity test was performed to compare the phase of behavior change between baseline and postintervention [75].

#### Demographics

Information on age (years), sex (male, female, or other), duration of T2D diagnosis (years), and diabetes-related complications (retinopathy, nephropathy, or neuropathy) was obtained from the hospital’s electronic patient records. During the intake, height (cm) and weight (kg) were measured and used to calculate the BMI (kg/m^2^). Having a job (yes or no) was questioned during the intake. Medication use was determined by means of the medication overview from the pharmacy that participants brought with them to the intake.

### Ethics Approval

The study protocol was approved by the Medical Ethics Research Committee Twente, Enschede (approval K20-05). In addition, the local advisory committee of practical feasibility at ZGT Hospital approved the study (approval ZGT17-39).

## Results

### Participant Characteristics

The 20 participants had a mean age of 68 (SD 8.0) years, BMI of 33.8 (SD 6.8) kg/m^2^, and T2D diagnosis of 19.7 (SD 9.8) years. The patient characteristics are presented in Table 1. In total, 15 participants evaluated the physical activity module, and 5 participants evaluated the nutritional module of E-Supporter 1.0.

**Table 1:** Participant characteristics (N=20).

Characteristic		Values
Age (years), mean (SD)		68.0 (8.0)
**Sex, n (%)**		
	Male	14 (70)
	Female	6 (30)
Weight (kg), mean (SD)		100.0 (21.2)
Length (cm), mean (SD)		172.0 (8.0)
BMI (kg/m^2^), mean (SD)		33.8 (6.8)
T2D^a^ duration (years), mean (SD)		19.7 (9.8)
**Complications, n (%)**		14 (70)^b^
	Retinopathy	3 (15)
	Neuropathy	7 (35)
	Nephropathy	6 (30)
Insulin treatment, n (%)		12 (60)
Employed, n (%)		7 (35)

^a^T2D: type 2 diabetes.

^b^The sum of the various complications (retinopathy, neuropathy, and nephropathy) is greater than the sum of complications because participants may experience multiple complications.

### Acceptability

#### Themes and Codes

The themes and main codes resulting from the interviews are provided in Table 2.

**Table 2:** Themes and codes resulting from the interviews.

Theme and main codes		Definition
**Perceptions regarding remote lifestyle coaching**		
	Continuous support in daily life	Opinions on the possibility of providing continuous and intensive lifestyle guidance through eHealth tools as E-Supporter 1.0
	Blended coaching	Beliefs about the application of E-Supporter as a blended-care intervention
**Perceptions regarding the coaching content**		
	Relevance of information	Opinions on the relevance of the content of E-Supporter 1.0
	Degree of tailoring	Perceptions about the degree of tailoring of the E-Supporter 1.0 content
**Intervention intensity and duration**		
	Use over time	Perceptions regarding the continued use of E-Supporter 1.0 over time
	Supporting behavior maintenance	Beliefs on whether E-Supporter 1.0 can adequately support behavior maintenance
**Perceived effectiveness**		
	Motivational effect	Perceptions about the motivational effect of E-Supporter 1.0 to change lifestyle behaviors
	Behavior change support	Opinions on whether E-Supporter 1.0 can realize lifestyle changes
**Overall appreciation**		
	Rating E-Supporter 1.0	Rating of E-Supporter 1.0 expressed on a scale from 1 to 10

#### Perceptions Regarding Remote Lifestyle Guidance

Many participants had positive opinions on the use of remote lifestyle coaching offered by E-Supporter 1.0. It was appreciated that daily guidance was provided. Due to continuous support, many participants expected that eHealth could guide people just as well or better than through face-to-face support. Nevertheless, some participants believed that digital lifestyle coaching would be less effective than intensive guidance from a health care professional during face-to-face consultations.

Participants preferred to use E-Supporter as a blended-care intervention in which they were supported by E-Supporter in addition to the consultations with their health care professional. One participant shared his preference of using eHealth over face-to-face consultations:

I will soon have an appointment with a dietician. If I had to choose between a dietitian or this, I would do this. I’ll be honest about that...Well, you know, you get a daily reminder. And with a dietician, a week before you go to the dietician, you think oh, let’s start with the advices. And I find that very difficult. This [E-Supporter] is a daily reminder, a text message, that makes you think: oh yes, what shall I have dinner tonight. Well then, I make something light with a lot of vegetables, or a light pasta meal, things like that.

Male participant, aged 59 years

#### Perceptions Regarding the Coaching Content

Participants were generally positive about the content of the motivational messages and emails. The practical tips on how to improve the lifestyle—generally messages with a call to action—were particularly appreciated. Participants who mainly received motivational messages targeting the initiation phase found the content aimed at increasing their knowledge to be logical, already known, or repetitive. Some participants used external information sources (eg, links to websites or videos) incorporated in the messages. Many participants thought that the external information sources were a good addition because they provided more in-depth information or advice on certain topics. Almost all participants were positive about the content of weekly emails with feedback on their behavior. The content of the emails was considered clear, relevant, and concise, as the participants responded as follows:

The email messages were very good in my opinion. They were very relevant and clear. So yes, I was satisfied with it. It gives you good insight into your progress and what you still need to work on.

Male participant, aged 57 years

Still, some participants felt that the email messages contained too much information, for example, regarding numerical information that was difficult for them to interpret.

The degree of tailoring in the messages was mainly experienced by the use of the participants’ first names. The content of the motivational messages themselves was not perceived as highly tailored. Most participants indicated that some messages matched their personal preferences regarding physical activities and diet, whereas other messages did not. According to the participants, the messages did not take sufficient consideration of personal factors (eg, physical limitations and mood) and external circumstances (eg, bad weather and working time). As the feedback was tailored to the participants’ behavior and their lifestyle goals, emails with feedback were generally considered more tailored than motivational messages.

#### Intervention Intensity and Duration

Generally, participants were satisfied with the intensity and duration of E-Supporter 1.0. The frequency of 2 motivational messages per day was considered sufficient by most participants. According to them, this frequency was sufficient to remind participants of a healthy lifestyle but not disturbingly. Some participants thought that the number of text messages per day was too high, as they could not follow all advice in this short period. The participants found a frequency of once a week for the emails to be just enough to receive an overview of their performance without being overwhelmed by information.

During the midintervention interviews, all participants indicated that they were still using E-Supporter. During the postintervention interviews, a vast majority of participants considered a length of 9 weeks to be a suitable time frame for lifestyle coaching via digital means. However, some participants indicated 9 weeks as a long period and therefore found it difficult to continue to actively use E-Supporter 1.0, especially in the final weeks. This was mainly caused by the repetitive nature of motivational messages and lack of interactivity in the coaching content. Nevertheless, other participants felt that the coaching period was too short to permanently change someone’s lifestyle and that longer guidance was needed to form new habits. Participant 19 commented on this perception as follows:

See, lifestyle patterns change, you have to motivate yourself, you have to get used to it. Nine weeks is too short. I am now used to eating less salty food and I am used to drinking less sugar. But every now and then, when I go out, I forget it again and relapse. And I think if you offer a slightly longer module, maybe 6 months, it will help many people a lot. Because people will be remembered every time until they get used to it [new lifestyle habits]. Then you adjust your lifestyle pattern.

Male participant, aged 59 years

#### Perceived Effectiveness

Participants found E-Supporter 1.0 to be a suitable way to gain more insight into their lifestyles.

The use of E-Supporter 1.0 increased their motivation to change their lifestyles because of the concrete tips and advice provided in the coaching content. Some participants indicated that the advice provided by E-Supporter helped them improve their lifestyle and health, which was illustrated as follows:

The benefit for me is that I have been more aware of a few things. Especially with the salt. I cook every day, so I think oh, potatoes salt, oh no, let’s not do salt. After 2 weeks I got used to it a bit. I had to have salt on everything and I have stopped doing that and that will help yourself...I have literally stopped drinking all soft drinks that contain sugar. Everything is zero or light. Those are those things, which is an added value for me. And I have already lost 3 kilos in that month and a half, so that is also good.

Male participant, aged 57 years

I was at the doctor this morning and he was so satisfied. My glucose was 2 points lower and my saturation was increased by 3% [chronic obstructive pulmonary disease as comorbidity]. Now I have experienced how good physical activity is.

Female participant, aged 62 years

However, other participants indicated that they did not find E-Supporter an added value for themselves but rather for people who are not yet planning to change their lifestyle.

#### Overall Appreciation

Table 3 provides an overview of the mean (SD) of the grades given for the various components of E-Supporter. All individual components of the modules for physical activity and healthy nutrition received a satisfactory mark from each participant, ranging from 7 to 9. E-Supporter 1.0 received an average overall rating of 8.0 (SD 0.5). Participants indicated that the biggest areas for improvement would be further tailoring of motivational messages and emails so that they better match someone’s personal and external circumstances.

**Table 3:** Mean (SD) grades (scale from 1 to 10) for the E-Supporter 1.0 modules and components.

Content	E-supporter, physical activity	E-supporter, healthy nutrition	E-supporter 1.0^a^
Motivational messages, mean (SD)	7.6 (1.1)	8.4 (0.5)	7.8 (1.0)
Feedback, mean (SD)	7.9 (0.5)	8.1 (0.2)	8.0 (0.5)
E-Supporter overall, mean (SD)	8.0 (0.5)	8.1 (0.2)	8.0 (0.5)

^a^Average of the E-Supporter grades for physical activity and healthy nutrition.

### Limited Efficacy

#### Physical Activity Levels

From baseline to postintervention, 11 of the 14 participants had increased their mean daily step count. Three participants had a lower mean daily step count postintervention than at the baseline. Owing to a Fitbit malfunction, we had missing data of 1 participant at baseline. The median daily step count was 6426.0 (IQR 2908.5-6811.5) steps per day at baseline. At midintervention, the median daily step count increased not significantly to 6988.5 (IQR 3424.5-9689.0) steps per day (*z*=−1.789; *P=*.07). Relative to baseline, there was a significant improvement in the daily step count to a median value of 8131.0 (IQR 4368.25-9855.5) steps per day postintervention (*z*=−2.040; *P=*.04).

#### Dietary Habits

The DHD15 index was calculated at baseline and postintervention for 2 of the 5 participants in the healthy diet module because the other participants did not complete the questionnaire postintervention. Both participants showed improvement in their adherence to the Dutch dietary guidelines. Participants 17 and 18 improved from a baseline score of 62.6 and 55.6 to scores of 77.9 and 68.9 postintervention, which are percent improvements of 24.4% and 22.3%, respectively. The dietary guidelines that the participants mainly improved on were an increase in the consumption of fish, nuts, and legumes and a decrease in the consumption of soft drinks and fruit juices.

#### Self-Efficacy Levels

Table 4 provides an overview of the self-efficacy levels of the participants at the baseline and postintervention. The third column in Table 4 shows the combined results for physical activity and health nutrition. At baseline, participants had a self-efficacy level with a median value of 33.5 (IQR 29.25-37.75). This self-efficacy level indicates that participants already had a high level of confidence in their own abilities to change behavior at baseline, using the cutoff value of Bay et al [72], where a score ≥29 is considered a high self-efficacy level. Overall, participants had a significantly improved self-efficacy level (median 33.5 [IQR 29.25-37.75] vs median 36.0 [IQR 34.0-37.0]) between baseline and postintervention (*z*=−1.997; *P=*.046).

**Table 4:** Self-efficacy levels and phase of behavior change at baseline (T0) and postintervention (T2) for the E-Supporter modules.

			E-supporter PA^a^					E-supporter NU^b^					E-supporter PA and NU	
			T0 (n=15)		T2 (n=12)^c^		T0 (n=5)			T2 (n=5)		T0 (n=20)		T2 (n=17)
Self-efficacy level, median (IQR)			34.0 (30.0-39.0)		36.0 (31.5-37.0)		33.0 (28.0-33.5)			37.0 (36.0-38.5)		33.5 (29.25-37.75)		36.0 (34.0-37.0)
**Phase of behavior change, n (%)**														
	Initiation	8 (53)		3 (25)		0 (0)			0 (0)		8 (40)			3 (18)
	Action	0 (0)		1 (8)		1 (20)			1 (20)		1 (5)			2 (12)
	Maintenance	7 (47)		8 (67)		4 (80)			4 (80)		11 (55)			12 (71)

^a^PA: physical activity.

^b^NU: nutrition.

^c^Missing data of 3 participants at T2.

#### Phase of Behavior Change

For the physical activity module, 8 participants were in the initiation phase, and 7 participants were in the maintenance phase at baseline (Table 4). At the postintervention assessment, we had missing data for 3 participants. A total of 3 participants in the initiation phase at baseline improved to a higher phase postintervention. Participants in the maintenance phase could not improve because this phase is already the highest. Participants in the nutritional module were already in the higher phases of behavioral change at baseline, including 1 participant in the action phase and 4 participants in the maintenance phase. The phase of behavior change of these participants remained unchanged postintervention. No significant improvement in the phase of behavior change was found between baseline and postintervention for the participants in either module (*P*=.17).

## Discussion

### Principal Findings

This study explored the acceptability of E-Supporter 1.0 among people with T2D and its limited efficacy in changing physical activity levels, dietary patterns, self-efficacy levels, and phase of behavior change in 9 weeks. E-Supporter was generally perceived as a motivating and potentially useful intervention to support lifestyle change, although the degree of tailoring was as yet modest. After the intervention period of 9 weeks, statistically significant improvements were found in steps per day and self-efficacy levels. Dietary patterns have improved the guidelines regarding the consumption of oily fish, nuts, legumes, and soft drinks and fruit juices. The behavior change phase remained almost unchanged.

E-Supporter 1.0 appeared to be a generally accepted intervention. Participants experienced the frequency of coaching and content as motivating and supportive of changing lifestyle behaviors. The various components (ie, motivational messages and feedback) and modules (ie, physical activity and nutrition) of E-Supporter 1.0 received more than sufficient grades for overall appreciation. We did not observe any major differences between the E-Supporter components and modules; therefore, it can be assumed that they are equally acceptable. These findings are encouraging because the acceptability of eHealth interventions among the target group is an important prerequisite for intervention engagement and effectiveness [54,55]. For accepted interventions, there is a greater chance that the intervention will be used as intended and therefore also have the desired effects [76,77]. This study showed that E-Supporter seems acceptable among an older target group, aligning with findings from previous studies, indicating that eHealth interventions are generally well received by older individuals when accompanied by suitable guidance [78,79]. The intervention’s relative simplicity, delivered through text messaging and email, may have contributed to its acceptance. This suggests that the intervention approach, provided that the intervention content is adapted to the needs of other target groups, could potentially be feasible for other target groups with limited technological affinity.

Overall, the degree of tailoring of the intervention was the item that participants were least satisfied with. E-Supporter focuses on different tailoring variables, consisting of behavior goals, phase of behavior change, chronic disease, time of day, and goal achievement. Some of these variables, such as chronic diseases, did not vary with time. In addition, tailoring to the phase of behavioral change may be difficult to recognize. Participants interpreted tailoring as the extent to which the content matched their daily activities, emotions, or environment. As E-Supporter does not yet focus on this type of time-dynamic variables [25], the degree of tailoring was experienced as limited. Improving the degree of tailoring will be an important target in the further development of E-Supporter, as this is important according to the participants and can also contribute to intervention effectiveness [51,52].

Some participants raised the question of whether an intervention period of 9 weeks is sufficient to permanently change behavior. How long it takes to successfully maintain a new behavior depends on several factors, such as the self-regulation skills of a person [80], their (social) living environment [81,82], the complexity of the behavior to be changed, or the time it takes to form habits [80]. The literature showed that it takes an average of more than 2 months (66 days) for a new behavior to become a habit [83]. The time frame required to form a habit varied widely per person from 18 to 254 days. Given the findings of the aforementioned studies, we expect that a tailored approach in terms of intervention support and duration may be valuable for providing each person with the support they need. Striking a balance between offering adequate support for relevant determinants while maintaining participants’ engagement to the intervention holds significance.

The secondary aim of this study was to explore whether this preliminary version of E-Supporter already has effects on physical activity, diet, self-efficacy levels, and the phase of behavioral change. Indeed, the results already showed some positive effects on the study participants. The median daily step count increased significantly by 1.705 steps per day postintervention compared with baseline. In addition, we found a significant improvement in self-efficacy level postintervention, despite an already high self-efficacy level at baseline. Moreover, 2 participants showed an improved diet according to the Dutch nutritional guidelines. No significant improvement was observed in the behavior change. This result can be explained by the fact that most participants were already in the maintenance phase at baseline, which means that no improvement was possible. However, the extent to which participants accurately assessed their behavioral change phase remains uncertain and whether their self-perception aligns with their actual lifestyle behavior, considering previous findings, indicates a tendency for individuals with T2D to overestimate their adherence to healthy behaviors [84]. To address this limitation, it would be advantageous to assess the phase of behavior change based on objective lifestyle data such as steps taken or nutritional information.

In our opinion, limited efficacy testing was an appropriate study aim for the stage of development that E-Supporter is in, namely, the first version of an intervention that still needs to be improved further [85]. This study showed initial positive findings that offer the potential for further development. However, no statements can be made regarding the effectiveness of E-Supporter. Without a control group, it is also difficult to determine whether the effects can be attributed entirely to the use of E-Supporter or to what extent the effects were caused by participation in the study in general. Most participants cooperated in the research because of their preexisting desire to improve their lifestyle. They perceived E-Supporter as a novel tool that could assist them in achieving this goal. Many participants were motivated by their desire to contribute to scientific research. These reasons inherently establish a population that is intrinsically motivated to actively engage in the study, potentially yielding better outcomes compared with individuals lacking intrinsic motivation. When E-Supporter is a more mature intervention with a more sophisticated degree of tailoring, its effectiveness will have to be investigated in larger studies with a control group.

### Strengths and Limitations

E-Supporter is developed iteratively on a scientific basis, together with end users [53]. A strength is that we evaluated E-Supporter between development iterations to identify key areas for improvement that will direct the further development of E-Supporter. A variety of aspects regarding the acceptability and limited efficacy on lifestyle behaviors were considered in this study, providing valuable first insights into the potential and areas for improvement of E-Supporter 1.0.

The sample size of this study was too small to make generalizable statements about the acceptability and effectiveness of E-Supporter 1.0. Nevertheless, we expect that this study provides a reasonable indication of the acceptability of E-Supporter because the intervention has been developed with end users. We have already ensured that the needs of the target group have been considered during development. In addition, limited efficacy testing was valuable to explore the first effects of E-Supporter and to examine whether it is useful to investigate the intervention in a larger-scale study [55]. The participants in this study were older adults with advanced diabetes. Therefore, these findings cannot be extrapolated to younger people with less advanced diabetes. In addition, the vast majority of participants were already in the maintenance phase of behavior change and already had a high self-efficacy level at baseline. As a result, we have only gained very limited insight into the acceptability and limited efficacy for people who have no intention of making lifestyle changes or who have little belief in their own ability to make lifestyle changes [86-88].

### Future Work

In this study, we identified concrete points to improve E-Supporter. We will aim to better support behavior maintenance and improve the degree of tailoring with regard to personal and environmental factors that influence lifestyle choices in future versions of E-Supporter. In the future, we will work toward an intervention that is more attuned to time-varying factors regarding the individual and their context. Hence, we strive to develop a *just-in-time adaptive intervention* that aims to provide the right support at the right time by adapting to an individual’s internal and contextual states [25]. A just-in-time adaptive intervention permits better tailoring of support to an individual’s real-time needs and can increase the sense of relevance of the support.

This study explored the limited efficacy of E-Supporter 1.0 and gave valuable insights for further development. This study primarily targeted an older population with advanced diabetes, as it was conducted in a hospital setting in which such individuals are predominantly treated. However, it is crucial to explore the feasibility of this approach in younger individuals who have recently been diagnosed with T2D. Conducting further research on this population is valuable, as adopting a healthy lifestyle can yield significant benefits in this particular group [89]. To be able to make statements about the effectiveness of E-Supporter 1.0 (or subsequent versions), studies with a larger sample size and control condition are needed in the future.

### Conclusions

In this exploratory study, E-Supporter already showed to be an acceptable and potentially useful intervention to promote physical activity and healthy dietary habits in people with T2D. Further work needs to be done to improve the degree of tailoring in E-Supporter 1.0 by adding just-in-time adaptive intervention content and to evaluate its effects in studies with a larger sample size and control condition.

## Appendix

Multimedia Appendix 1 Interview schedule E-Supporter acceptability.

Multimedia Appendix 2 Self-efficacy questionnaires.

Multimedia Appendix 3 Single-question SAS stages of change.

## Abbreviations

BCT: behavior change technique
DHD15 index: Dutch Healthy Diet index
DIALECT: Diabetes and Lifestyle Cohort Twente
ESES: Exercise Self-Efficacy Scale
FFQ: Food Frequency Questionnaire
SAS: Self-Assessment Scale
T2D: type 2 diabetes
ZGT: Ziekenhuisgroep Twente

## Data Availability

The data generated during this study are not publicly available because of privacy restrictions but are available from the corresponding author on reasonable request via the DANS EASY repository [90].
