# Supplementary material for: A Digital Coach (E-Supporter 1.0) to Support Physical Activity and a Healthy Diet in People With Type 2 Diabetes: Acceptability and Limited Efficacy Testing
Source: JMIR Form Res. 2023 Jul 28;7:e45294. doi: 10.2196/45294 (PMC10422172; doi:10.2196/45294)
Supplement: Multimedia Appendix 1 [file formative_v7i1e45294_app1.pdf]

Multimedia Appendix 1. Interview schedule E-Supporter acceptability

Introduction

Hello, You speak to <*name of researcher*>, the researcher from the hospital in Almelo. I am calling you because we have scheduled the interview for today. With this interview we want to gain more insight into what you thought of the coaching via your mobile phone.

The interview consists of three parts, in which I would like to ask some questions about the following topics:

- The coaching module in general;
- The SMS messages you have received;
- The e-mails you have received;

If you agree, I will record this interview in an audio clip, so that I can listen to your answers afterwards. As described in the study information, these audio recordings will be used only for the purposes of this study. Do you still agree with this? Do you have any questions for me before we start the interview?

If you have no more questions for now, we can start with the interview with the aim of hearing what you thought of the coaching module.

START AUDIO-RECORDING

Topic 1: General appreciation of the intervention

- What do you think of the coaching module?
- Do you think that the coaching module is in line with your needs for a <*physical activity/healthy diet*>?
- Do you think that the coaching module can support you to improve your <*physical activity/ diet*>?
  - Could you elaborate on why you expect the the coaching module (not) to be helpful?
- What do you think of the length of the coaching module?
- Do you find the videos, which you could find with a link in the text messages, of added value?
- Do you find the links to websites in the text messages of added value?
- What do you think of this way of remote healthcare?
- Would you like this remote contact instead of a real life appointment to improve your <*physical activity/healthy diet*>?

Topic 2: Motivational messages

- What do you think about the frequency of the text messages?
- What do you think of the content of the text messages?
  - Could you give an example of a message with good content?
  - Could you give an example of a message with less good content?
- Do you consider the text messages personal?
  - How do you notice this?/ What would you improve?
- Do you think the text messages are tailored to your personal wishes and goals?
  - How do you notice this?
- Did you miss anything in the text messages?
- How would you like to improve the text messages?

Topic 3: Feedback

- What do you think about the frequency of the e-mails?
- What do you think of the content of the e-mail messages?
    - What did you like?
    - What did you think was less good?
- What do you think of the e-mail messages in addition to the text messages?
- Do you consider the e-mail messages personal?
    - How do you notice this?/ What would you improve?
- Did you miss something in the email messages?
- How would you like to improve the email messages?

Topic 4: Grading the E-Supporter

What grade would you rate the E-Supporter on a scale of 1 to 10? A 1 means you thought it was very bad and a 10 indicates you thought it was excellent. I am going to ask you for a separate grade for each part and whether you could briefly explain this grade.

- SMS:
- Email:
- Overall

Closing

Those were all the questions I had for you.

- Do you have any additional comments about your experience with the coaching module that have not been addressed in the questions?
- Do you have any questions?

Then this is the end of the interview. I would like to thank you for participating in the study and sharing your opinion. Thank you very much for your time and have a nice day.
