# Supplementary material for: A Digital Coach (E-Supporter 1.0) to Support Physical Activity and a Healthy Diet in People With Type 2 Diabetes: Acceptability and Limited Efficacy Testing
Source: JMIR Form Res. 2023 Jul 28;7:e45294. doi: 10.2196/45294 (PMC10422172; doi:10.2196/45294)
Supplement: Multimedia Appendix 2 [file formative_v7i1e45294_app2.pdf]

Multimedia Appendix 2. Self-efficacy questionnaires

EXERCISE SELF-EFFICACY SCALE (ESES) [57]

We ask you to indicate how confident you are in your ability to move. Exercise is understood to mean both everyday physical activity (for example walking, cycling, wheelchair driving or hand bike for walking the dog or shopping) and sports (for example fitness or swimming).

(Please tick only 1 box per question)

I am convinced:	Not true at all	Rarely true	Sometimes true	Always true
That I can overcome barriers and challenges with regard to physical activity and exercise if I try hard enough	☐	☐	☐	☐
That I can find means and ways to be physically active and exercise	☐	☐	☐	☐
That I can accomplish my physical activity and exercise goals that I set	☐	☐	☐	☐
That when I am confronted with a barrier to physical activity or exercise I can find several solutions to overcome this barrier	☐	☐	☐	☐
That I can be physically active or exercise even when I am tired	☐	☐	☐	☐
That I can be physically active or exercise even when I am feeling depressed	☐	☐	☐	☐
That I can be physically active or exercise even without the support of my family or friends	☐	☐	☐	☐
That I can be physically active or exercise without the help of a therapist or trainer	☐	☐	☐	☐
That I can motivate myself to start being physically active or exercising again after I've stopped for a while	☐	☐	☐	☐
That I can be physically active or exercise even if I had no access to a gym, exercise, training or rehabilitation facility	☐	☐	☐	☐

HEALTHY EATING SELF-EFFICACY SCALE (adapted version by Fokkema [71])

We ask you to indicate how confident you are in your ability to eat healthier. This includes eating more fruit and vegetables, eating less sugar, eating smaller ones eat portions, pay attention to carbohydrates, et cetera.

(Please tick only 1 box per question)

I am convinced:	Not true at all	Rarely true	Sometimes true	Always true
That I can overcome barriers and challenges with regard to healthy eating if I try hard enough	☐	☐	☐	☐
That I can find means and ways to eat healthy.	☐	☐	☐	☐
That I can accomplish my healthy eating goals that I set	☐	☐	☐	☐
That when I am confronted with a barrier to healthy eating I can find several solutions to overcome this barrier	☐	☐	☐	☐
That I can eat healthy even when I am tired	☐	☐	☐	☐
That I can eat healthy even when I am feeling depressed	☐	☐	☐	☐
That I can eat healthy even without the support of my family or friends	☐	☐	☐	☐
That I can eat healthy without the help of a dietician or other professional	☐	☐	☐	☐
That I can motivate myself to start eating healthy again after I’ve stopped for a while	☐	☐	☐	☐
That I can eat healthy even if it means I have to cook this myself	☐	☐	☐	☐
