# Supplementary material for: A Digital Coach (E-Supporter 1.0) to Support Physical Activity and a Healthy Diet in People With Type 2 Diabetes: Acceptability and Limited Efficacy Testing
Source: JMIR Form Res. 2023 Jul 28;7:e45294. doi: 10.2196/45294 (PMC10422172; doi:10.2196/45294)
Supplement: Multimedia Appendix 3 [file formative_v7i1e45294_app3.pdf]

Multimedia Appendix 3. Single-question SAS Stages of Change

Phase of behavior change questionnaire physical activity [58]

Please tick the answer that applies to you for the question below. Only one answer is possible. Are you physically active (walking, cycling, or exercising) for more than 30 minutes each time at least 5 days a week?

- ❒ Yes, and I have been that for more than 6 months
- ❒ Yes, but I only started this in the last 6 months
- ❒ No, but I plan to do so in the next 30 days
- ❒ No, but I plan to do so in the next 6 months
- ❒ No, and I don't intend to in the next 6 months

Phase of behavior change questionnaire nutrition (adapted version by Fokkema [71])

Please tick the answer that applies to you for the question below. Only one answer is possible. Are you consciously engaged in healthy eating for most of the day (for example, eating less carbohydrates, eating enough vegetables and fruit)?

- ❒ Yes, and I have been that for more than 6 months
- ❒ Yes, but I only started doing that in the last 6 months
- ❒ No, but I plan to do so in the next 30 days
- ❒ No, but I plan to do so in the next 6 months
- ❒ No, and I don't intend to in the next 6 months
